# Phosphatidylserine, inflammation, and central nervous system diseases

**DOI:** 10.3389/fnagi.2022.975176

**Published:** 2022-08-03

**Authors:** Xiaohua Ma, Xiaojing Li, Wenjuan Wang, Meng Zhang, Bo Yang, Zhigang Miao

**Affiliations:** ^1^Department of Neurology and Clinical Research Center of Neurological Disease, The Second Affiliated Hospital of Soochow University, Suzhou, China; ^2^Institute of Neuroscience, Soochow University, Suzhou, China; ^3^Suzhou Science and Technology Town Hospital, Suzhou, China; ^4^Department of Anesthesiology, The Second Affiliated Hospital of Soochow University, Suzhou, China

**Keywords:** phosphatidylserine, phosphatidylserine liposomes, neurotransmission, synaptic refinement, central nervous system disease

## Abstract

Phosphatidylserine (PS) is an anionic phospholipid in the eukaryotic membrane and is abundant in the brain. Accumulated studies have revealed that PS is involved in the multiple functions of the brain, such as activation of membrane signaling pathways, neuroinflammation, neurotransmission, and synaptic refinement. Those functions of PS are related to central nervous system (CNS) diseases. In this review, we discuss the metabolism of PS, the anti-inflammation function of PS in the brain; the alterations of PS in different CNS diseases, and the possibility of PS to serve as a therapeutic agent for diseases. Clinical studies have showed that PS has no side effects and is well tolerated. Therefore, PS and PS liposome could be a promising supplementation for these neurodegenerative and neurodevelopmental diseases.

## Introduction

Phosphatidylserine (PS) is a structural component of the eukaryotic membrane and is accounted for 5–10% of the total lipid of cells (van Meer et al., 2008; Vance, 2015). Given its unique physical and biochemical properties as an anionic phospholipid, PS binds to various proteins and is involved in many biological processes, including enzyme activation, apoptosis, neurotransmission, and synaptic refinement (Fadok et al., 1992; Zhang et al., 2009; Huang et al., 2011; Scott-Hewitt et al., 2020). Therefore, the dysregulation on the metabolism of PS is associated with different CNS diseases, including Alzheimer’s disease (AD), Parkinson’s disease (PD), major depressive disorder (MDD), stroke, and autism spectrum disease (ASD) (Enseleit et al., 1984; Fabelo et al., 2011; El-Ansary et al., 2016; Tokuoka et al., 2019; Homorogan et al., 2021). In addition, PS supplementation is proved to benefit the patients with AD, MDD, PD, or ADHD (Funfgeld et al., 1989; Maggioni et al., 1990; Crook et al., 1992; Hirayama et al., 2014). Chronic neuroinflammation is implicated in these CNS diseases, PS supplementation can inhibit excessive neuroinflammation to play a neuroprotective role. Moreover, PS supplementation can improve the cognitive function of the brain. In this review, we summarized the role of PS in the brain and its role in several related CNS diseases.

## The biosynthesis, distribution, asymmetry, and degradation of phosphatidylserine

**FIGURE 1:**
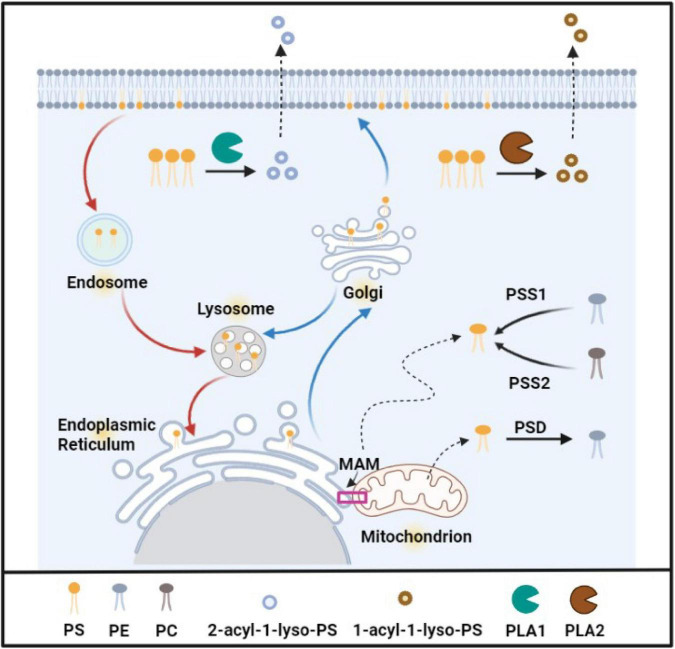
The biosynthesis, distribution, and degradation of phosphatidylserine. PS is produced in ER (MAM), PSS1 catalyzes PE to PS, PSS2 catalyzes PC to PS. Some new PS is transported to mitochondria, and PS is decarboxylased and forms PE in mitochondria. PS is also transferred to the plasma membrane and other oranges by the Golgi via traditional vesicle-mediated trafficking. PS in the endosome is recycled to the plasma membrane. PS in lysosomes is derived from the Golgi apparatus or endosome. PS can be hydrolyzed by phospholipase A1 (PLA1) and phospholipase A2 (PLA2), the productions are 2-acyl-1-lyso -PS and 1-acyl-2-lyso-PS, respectively. Created with BioRender.com.

As an important glycerophospholipid, PS was first identified in the whole-brain lipid extracts in the 1940s (Folch, 1948). Its glycerol moiety contains two acyl chains at the *sn-1* and *sn-2* positions and a polar-head group at position *sn-3*, in which the neutral amino acid serine locates (Leventis and Grinstein, 2010). As shown in Figure 1, PS is produced by exchanging headgroups in mammalian cells by PS synthases; for example, PS synthase 1 is responsible for exchanging headgroup choline from PC (phosphatidylcholine), and PS synthase 2 is responsible for exchanging headgroup ethanolamine from PE (phosphatidylethanolamine). Because PS synthase 1 and 2 are uniquely expressed in the mitochondrial-associated membranes (MAMs) of the endoplasmic reticulum, PS is produced in the endoplasmic reticulum and transferred to the mitochondria or the Golgi through MAMs (Stone and Vance, 2000). In the mitochondria, a part of PS is catalyzed to PE by PS decarboxylase in the inner leaflet of mitochondria, while the other part of PS is incorporated into the mitochondrial membrane (Camici and Corazzi, 1995). Some newly synthetic PS is transferred from the endoplasmic reticulum to the Golgi intermediate compartment and the Golgi cisternae via the secretory pathway (Voelker, 2000), then PS is secreted to the plasma membrane or is delivered to the endosome and the lysosome. PS in the endosome, especially recycling endosomes, is slowly recycled to the plasma membrane (Voelker, 2000). In the normal conditions, PS is located exclusively in the cytoplasmic leaflet of the plasma membrane, endoplasmic reticulum lumen, Golgi, mitochondria, and endosomes to maintain the normal function of organelles (Yeung et al., 2008; Leventis and Grinstein, 2010; Kay and Fairn, 2019). The detailed biological events of PS and the percentage of PS in total phospholipid in different organelles were summarized and listed as in Table 1.

**TABLE 1:** The biological events of phosphatidylserine (PS) and the percentage of PS in total phospholipid of different organelles.

Organelles membrane	PS%	The key protein	Events of PS distribution and metabolism
Plasma membrane	12	Flippase Floppase Scramblase	Flippase transports PS from the extracellular to the cytosolic side, floppase transports PS from the cytosolic to the extracellular side, scramblases transports PS bidirectionally.
Endoplasmic reticulum	3–5	PSS1 and PSS2 scramblases	Produce PS by PSS1 and PSS2 in MAMs, scramblases translocate PS synthesized on the cytosolic side to the internal leaflet.
Golgi complex	5	P4-ATPase	Keep PS asymmetry, transport PS to plasma membrane, divert PS to the prelysosomal endocytic compartment.
Early endosome	8.5	ATP8A1, ATP8A2, ATP9A, EHD1	ATP8A1, ATP8A2 and ATP9A are PS flippases, EHD1 is a PS effector, all of them are essential for endosomal traffic through recycling endosomes.
Late endosome	2.5–3.9		
mitochondria	1	PS decarboxylase	Decarboxylate PS to PE on the outer leaflet of the mitochondrial inner membrane

EHD1, Eps15 homology domain-containing protein 1.

Degradation of PS is carried out via two enzymes: PS decarboxylases and phospholipases (as shown in Figure 1). As previously described (Camici and Corazzi, 1995), PS decarboxylases catalyze PS to form PE in the mitochondria. There are two: PS-specific phospholipases A1 and A2. Both phospholipases catalyze a reaction to produce Lyso-phosphatidylserine (2-acyl-1-lyso-PS and 1-acyl-2-lyso-PS). PS-specific phospholipase A1 (PS-PLA1) hydrolyzes the *sn-1* acyl chain of PS exposed on the surface of cells such as apoptotic cells or activated platelets, and generates 2-acyl-1-lyso-PS which is a mediator for the activation of mast cells, T cells and neural cells (Wen et al., 2001). PS-specific phospholipase A2 (PS-PLA2) is also essential to inflammation and the immune response. It hydrolyzes the *sn-2* acyl of PS to produce 1-acyl-2-lyso-PS and further to form many bioactive lipid mediators in many biological processes (Funk, 2001). Therefore, lyso-phosphatidylserine is involved in a series of biological process such as apoptosis and T cell activation (Bellini and Bruni, 1993). For example, when PS is exposed during apoptosis, PS-PLA1 hydrolyzed PS on the cell surface and produces 1-acyl-2-lyso-PS, stimulates histamine release from mast cells in the presence of Fc*upvarepsilon*RI cross-linker, and induces inflammation and cell death (Hosono et al., 2001). In addition, Lyso-phosphatidylserine can also enhance nerve growth factor-induced neural differentiation, and may play a neuroprotective role to improve tissue restoration after brain damage occurs (Lourenssen and Blennerhassett, 1998).

**FIGURE 2:**
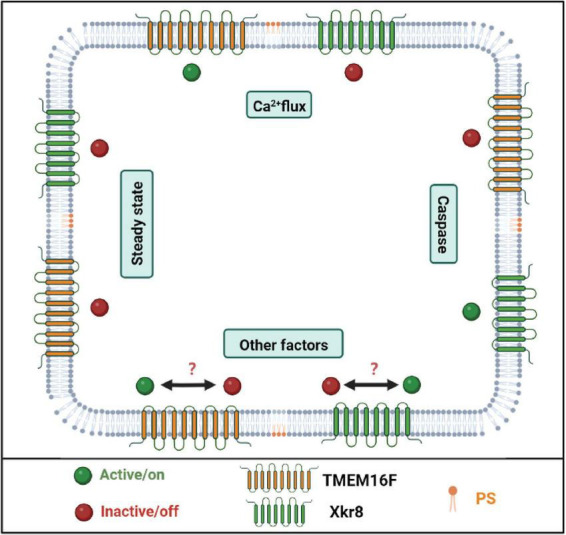
The scrambles are responsible for phosphatidylserine (PS) exposure. PS scrambles are responsible for PS translocation during cell apoptosis as well as other biological processes. TMEM16F and XKr8 are well-documented PS scrambles. When the intercellular Ca^2+^ concentration is upregulated, TMEM16F binds Ca^2+^ and transports PS to the outer leaflet of the plasma membrane. When the cell undergoes apoptosis, caspase cleaves and activates XKr8, and then XKr8 exposes PS to the outside of the cell and releases the “eat me” signal. But in other biological processes, it remains unknown which scramble is activated or both of them are activated. Created with BioRender.com.

To maintain normal cellular function, PS is distributed in the inner leaflet of the lipid bilayers of the membrane; otherwise, cells are induced to apoptosis as mentioned above when PS is exposed on the outer leaflet of the lipid bilayers (Chua et al., 2019). How is the distribution asymmetry of PS regulated in the cellular lipid bilayers? Flippase, floppases, and scramblases are three lipid transporter enzymes in the membranes that dictate the fate of PS distribution. The P4 subfamily of P-type ATPases (P4-ATPases) is identified as flippase which transport PS and other lipids from the extracellular to the cytosolic side of the membrane in an ATP-independent manner. All P4-ATPases are critical to minimize PS exposure. Total fourteen P4-ATPases are identified in the human genome; some of them are located in the plasma membrane (such as ATP11A and ATP11C), while some is located in the endosome membrane (such as ATP8A1, ATP8A2, and ATP9A) (Nagata et al., 2020). Most P4-ATPases require CDC50A (TMEM30A) as a functional subunit for target localization (Coleman and Molday, 2011). Deletion of the chaperone CDC50A in the cell promotes PS exposure and cellular engulfment by macrophage (Segawa et al., 2014). Opposite to flippase, floppase transports lipid from the cytosolic to the extracellular side of the membrane. Lipid floppases are identified as members of the ATP-binding cassette (ABC) transporter superfamily. Floppase ABCA1 is responsible for PS and cholesterol transportation and has been found to have a critical role in lipid efflux and plasma membrane remodeling (Gulshan et al., 2013). Scramblases are also important lipid transporters that transport PS bidirectionally in an ATP-independent manner (Leventis and Grinstein, 2010). Two family members TMEM16 and Xk-related (XKR) protein are identified to have scramblases activity (Suzuki et al., 2013; Kalienkova et al., 2021). Among the two families, TMEM16F and XKR 8 are well-documented scramblases. As shown in Figure 2, TMEM16F is a Ca^2+^-dependent scramblase, while XKR8 responds to the caspase signal (Hankins et al., 2015). However, these transporters may be interacted or crosstalk between them may regulate PS metabolism. For example, in the apoptotic cells or other biological processes, the flippases are disrupted by caspase or inhibited by Ca^2+^; at the same time, either TMEM16F or XKR8 is activated to expose PS and participate these biological processes (Nagata et al., 2020).

## The functions of endogenous phosphatidylserine

### As the protein docking sites on the cell membrane, phosphatidylserine participates in the activation of many signaling pathways

PS is the major acidic phospholipid in the human brain, accounting for 11.4–14.4% in the cerebral cortex, and 16.0–21.1% in white matter and myelin (Svennerholm, 1968; Horrocks, 1973). Hence, PS is an essential nutrient for the brain and is implicated in the normal functions of the brain. As the structural lipid in the cell membrane, many signaling molecules or proteins interact with PS through their C2 domain or gamma-carboxyglutamic acid domain in the presence of Ca^2+^; PS is located inside the cells and servers as to recruit and activate signal pathways, including PKC family, phosphoinositide-3-kinase (PI3K)/AKT and Ras/Raf. For example, protein kinase C (PKC) family members bind PS through their C2 domains, while growth arrest-specific 6 (Gas6) binds PS via gamma-carboxyglutamic acid domain (Rajotte et al., 2008). In addition, due to the negatively charged headgroup of PS, some proteins bind PS in a non-specific charge-based manner, such as protein kinase Src, Rac1, and K-Ras, and activate these kinase and their downstream signals (Stace and Ktistakis, 2006). These signal pathways have been well described to support neuronal cell survival, differentiation, and proliferation (Kim et al., 2014; Glade and Smith, 2015; Kay and Fairn, 2019).

### Phosphatidylserine is involved in neurotransmission

**FIGURE 3:**
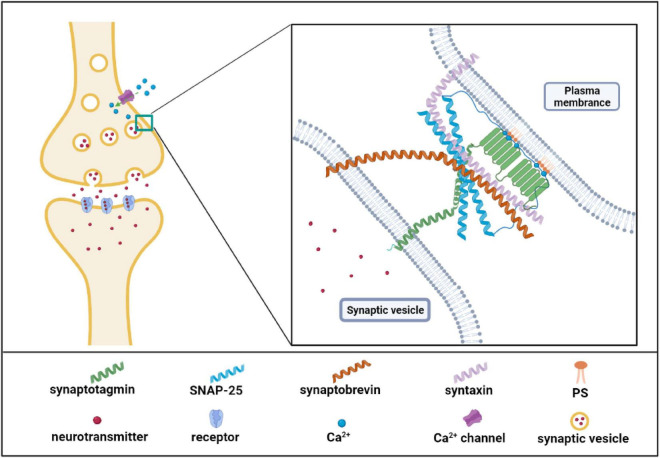
Phosphatidylserine (PS) effector protein synaptotagmin I is involved in neurotransmitter release. As the figure shows, synaptotagmin I possesses two C2 domains to bind Ca^2+^ and PS. N-terminus of synaptotagmin I is in synaptic vesicles and C2 domain in cytoplasmic. Synaptotagmin I binds Ca^2+^ when Ca^2+^ flows into cells from the Ca^2+^ channels. The Ca^2+^ binding further increases the binding of synaptotagmin I to SNARE complex as well as the fusion of synaptic and plasma membrane. SNARE complex includes the synaptosome-associated protein of 25 kDa (SNAP25), synataxin, and synaotobrevin. Created with BioRender.com.

Neurotransmission is a biological process by which neurons transmit information and maintain normal functions of the brain. Structurally, the neurotransmitters which are packaged in synaptic vesicles are released by presynaptic membranes via calcium-dependent exocytosis and then bind to the receptor in the postsynaptic membrane and complete the signal transmission between neurons (Kimura, 2021). PS is a component of the synaptic vesicle and involves several necessary neurotransmission steps. On the one hand, PS could directly bind to neurotransmitters, and the binding facilitated the availability of neurotransmitters for their re-uptake. For examples, PS has the highest affinity among acidic lipids to bind serotonin in brain tissues (Johnson et al., 1977); the headgroup of PS in lipid bilayer is strongly bound to dopamine or L-dopa through H-bonds (Orlowski et al., 2012). The interaction between PS and neurotransmitters may facilitate the uptake process or metabolization of neurotransmitters and exert their effects (Orlowski et al., 2012). On the other hand, synaptic vesicle exocytosis is the most important neurotransmission process. Previous studies showed that PS treatment increases synaptic vesicle number adjacent to the plasma membrane and upregulates the frequency of calcium-dependent exocytosis of synaptic vesicles (Uchiyama et al., 2007; Zhang et al., 2009). Synaptic vesicle exocytosis includes three critical steps: vesicle docking, prime, and fusion. PS may influence neurotransmitter vesicle docking and fusion at the plasma membrane. Recently a study demonstrated that PS promoted vesicle docking through interaction with α-synuclein (α-Syn). When PS levels in the vesicle membrane decrease, α-Syn inhibits the vesicle docking; however, high levels of PS can bind to α-synuclein and reverse its inhibition on the vesicle docking (Lou et al., 2017), suggesting the critical role of PS in α-Syn-mediated vesicle docking. Similarly, PS also regulates the opening and dilation of the fusion pore via interaction with synaptotagmin I (syt-1), the major calcium sensor for synaptic vesicle exocytosis (Brose et al., 1992). This process is shown in Figure 3, PS or phosphatidylinositol is able to bind the C2 domains of Syt-1 to promote the fusion of synaptic vesicle membrane with the cell membrane (Tucker et al., 2004; Gruget et al., 2020). Furthermore, the high level of PS increases the binding affinity of Syt-1 to *N*-ethylmaleimide-sensitive fusion protein receptor complex (SNARE) at the plasma membrane, which was the core machinery complex for exocytosis. The Syt-1/SNARE complex undergoes Ca^2+^- dependent oligomerized and inserts into the presynaptic membrane and facilitates the opening and dilation of the fusion pore for the neurotransmitter release (Hosono et al., 2001; Koh and Bellen, 2003; Zhang et al., 2009; Figure 3). In summary, PS is involved in the biological processes of the release and re-uptake of neurotransmitters.

### Phosphatidylserine is linked to glia-mediated synaptic refinement

Synaptic refinement, as known as synaptic pruning, is a process to eliminate supernumerary synapses. Impairment of synaptic pruning is involved in the pathogenesis of ASD, MDD, AD, and other mental conditions (Brown and Neher, 2014; Vilalta and Brown, 2018). Previous studies showed that microglia and astrocytes mediate the regulation of synaptic pruning (Schuldiner and Yaron, 2015; Wilton et al., 2019). In the developmental and adult brain, glia-mediated synaptic refinement is an essential physiological process to keep a proper number of synapses to maintain functional neuronal circuits (Schuldiner and Yaron, 2015). Similar to the apoptotic process, PS externalization in synapses takes part in glia-mediated synaptic pruning process in the developing hippocampus where there are abundant synapses that need to be pruned (Scott-Hewitt et al., 2020). On the contrary, microglial synapse elimination can be prevented by blocking accessibility of exposed PS using Annexin V or TREM2 loss in microglia (Scott-Hewitt et al., 2020). In addition, mitochondrial activity reduction contributes to PS exposure on axons, which is a marker of axonal pruning (Shacham-Silverberg et al., 2018).

How PS involved in glia-mediate synaptic refinement and axonal pruning is remains unclear? A previous study showed that the complement protein C1q was also involved in the synaptic pruning process by recognizing exposed PS in synapses (Scott-Hewitt et al., 2020). Deficiency of C1q in mice reduces the microglial engulfment of synapses and results in excessive retinal innervation of lateral geniculate neurons (Scott-Hewitt et al., 2020). The S4 variant of GPR56 splicing isoforms is also found to bind PS to mediate synaptic pruning by microglia; however, the deletion of GPR56 in microglia fails to bind exposed PS and leads to excess synapses (Li et al., 2020). In addition, some PS receptors that recognize exposed PS in synapses are considered to mediate synaptic pruning. Deletion of PS flippase chaperone CDC50A induces PS exposure on neuronal somas and specifically eliminates inhibitory post-synapse through microglial PS receptor Mer (Li et al., 2021). Previous studies also showed that PS receptors TREM2 and MEFG10 contribute to synaptic refinement in microglia (Filipello et al., 2018) and in astrocytes (Lee et al., 2021), respectively. Overall, PS exposure was a marker of unwanted synapses and axons; PS receptors bind to exposed PS and trigger synaptic pruning.

### Phosphatidylserine located in the outer of cell membrane is a marker of apoptosis

The increasing lines of evidence demonstrate that PS exposure in the outer leaflet of cell membrane can be caused by apoptosis including intrinsic apoptosis and extrinsic apoptosis (Kiraz et al., 2016). Intrinsic apoptosis is induced by mitochondrial stress and the release of cytochrome C from the mitochondria to the cytosol. Cytochrome C in cytosol activates apoptotic protease-activating factor 1 and caspase cascades like caspase 9 and caspases 3/7, resulting in intrinsic apoptosis (Segawa et al., 2014). Extrinsic apoptosis is mediated by death receptors including tumor necrosis factor receptor 1, the Fas receptor (CD95), and the tumor necrosis factor-related apoptosis-inducing ligand (traiL) receptors. Death receptors interact with their ligands to activate caspase 8, and activating caspase 8, in turn, activates caspase3/7. Activated caspase3/7 is a key to trigger PS exposure and cell apoptosis (Boada-Romero et al., 2020).

During apoptosis, caspases 3/7 inactivates PS flippase and activates PS scramblases to expose PS (Segawa et al., 2014). ATP11C (adenosine triphosphatase type 11C), a PS flippase family member, is essential to maintain PS asymmetry in the cell membrane (Takada et al., 2015; Segawa et al., 2018). Activated caspases 3/7 cleave ATP11C upon the recognized sites of ATP11C causing PS exposure in the outer of cell membrane (Segawa et al., 2014). PS exposure is abolished and cells are not induced to apoptosis and are not engulfed by macrophages when caspase recognized sites of ATP11C are mutated (Segawa et al., 2014). In addition, PS scramblases XKR8, which is important for PS asymmetry, is also cleaved by caspase3/7 upon the recognized site at C-terminus of XKR8 and activated (Suzuki et al., 2014). Active XKR8 forms a high-order complex with basigin and neuroplastin, two chaperone proteins of XKR8 (Suzuki et al., 2014), and then exposes PS to cell surface (Suzuki et al., 2016).

**FIGURE 4:**
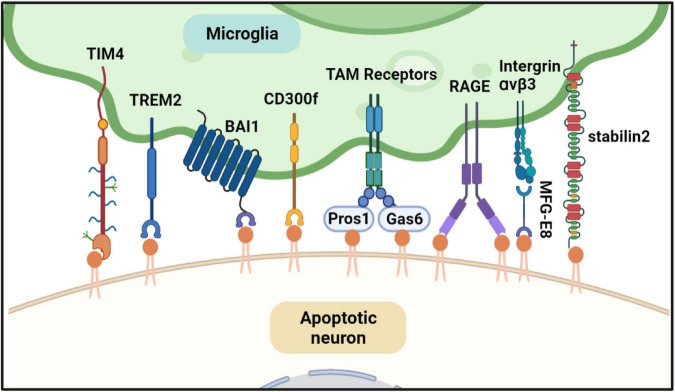
Phosphatidylserine (PS) is involved in microglia-mediated neuron apoptosis. When the neuron is undergoing apoptosis, PS is exposed to the cell surface. Microglia can find and phagocyte the apoptotic neuron. Several PS effectors expressed in microglia can recognize the exposed PS directly or indirectly. TREM2, RAGE, TIM4, and BAI1 can bind PS directly. TAM receptors recognize PS indirectly through their ligands Gas6 and ProS1. Both Gas6 and ProS1 have Gla-domain to bind PS. As well as TAM receptor, intergrinαvβ3 recognize PS through MFG-E8. All of the PS effectors can trigger cytoskeletal rearrangement of microglia and engulf the dying neuron. Created with BioRender.com.

Externalization of PS serves as a marker of apoptotic cells and an “eat me” signal. Exposed PS on the cell surface is recognized by PS receptors expressed in macrophages and further initiates actin reorganization to engulf apoptotic cells by macrophages (Lemke, 2019). PS receptors in macrophages that bind PS directly or indirectly are shown in Figure 4. Several PS receptors can recognize PS directly, such as T-cell immunoglobulin and mucin domain-containing molecule-1 and -4 (TIM-1, -4), receptor for advanced glycation end products (RAGE), brain-specific angiogenesis inhibitor 1 (BAI1), triggering receptor expressed on myeloid cells 2 (TREM2), stabilin 2, and members of the CD300 family (Park et al., 2007; Paidassi et al., 2008; Park et al., 2008; He et al., 2011; Tian et al., 2014; Krasemann et al., 2017). Other PS receptors, such as integrin αvβ3 and TAM receptors, bind to PS indirectly and need bridging molecules. For example, integrin αvβ3 binds to the epidermal growth factor domain of Milk fat globule-EGF factor 8, which is a soluble PS receptor to recognize exposed PS and apoptotic cells and mediate apoptotic signal (Fuller and Van Eldik, 2008); Protein S and growth arrest specific 6 also recognize PS and bridge TAM receptors with apoptotic cell (Lemke, 2017). Most PS receptors are expressed in peripheral macrophages and microglia, the primary tissue-resident macrophages in the brain (Nazareth et al., 2021).

## The function of exogenous phosphatidylserine

Exogenous PS could obtain from the bovine brain and krill, and also be made from soybean lecithin by the enzymatic reaction with L-serine (Glade and Smith, 2015). When exogenous PS is given, PS can be uptake and transported in the cell by PS flippase, then incorporated into the membrane system to support cell function (Glade and Smith, 2015). In addition, PS liposomes that exposed PS on the surface could mimic apoptotic cells, be recognized by PS receptors and engulfed by phagocytes, and then triggered anti-inflammatory signal pathways (Aramaki et al., 2001).

### Phosphatidylserine improves the cognitive function of the brain

Increasing studies have demonstrated that supplementation of PS significantly improved the cognitive impairment caused by aging, AD, or PD (Cenacchi et al., 1993; Kim et al., 2014; More et al., 2014). In a double-blind study, the elder patients with severe cognitive decline were treated with brain cortex-derived PS (BC-PS) 300 mg/day for 6 months, compared with placebos, BC-PS administration significantly improved the storage, learning, and retrieval abilities of memory in patients. Treatment the aged patients with BC-PS (300 mg/day) for 12 weeks also resulted in significant improvement of cognitive function (Schreiber et al., 2000). These results suggested that choric PS treatment ameliorates the cognitive function of the brain. Previous literatures demonstrate that PS benefits brain functions in several ways.

First, PS may improve function of brain through PKC. Since PKC activation influences the process of cognition through regulating phosphorylation of substrates such as *N*-methyl-D-aspartate (NMDA) receptor, AMPA receptor, and growth-associated protein-43 (Sun and Alkon, 2010), the expressions of several PKC isoforms are down-regulated in cognition impairment conditions, including aging, PD, and AD (Mizutani et al., 1998; Alkon et al., 2007; Do Van et al., 2016). Intake of exogenous PS could promote the activity of PKC (Bell and Burns, 1991), which phosphorylated its substrates to boost cell function (Sun and Alkon, 2010). Secondly, PS may ameliorate the cognitive function through enhancing glucose mentalism, which is related to cognitive impairment. Abnormal glucose metabolism rates of AD was found in predominantly disease-affected brain regions of patients with AD and other types of dementia (Friedland et al., 1983). The administration of exogenous PS (500 mg daily for 3 weeks) increased global glucose metabolism by 14.8% in the brain cortex and significantly improved the cognition of AD patients (Heiss et al., 1991; Klinkhammer et al., 1991). Thirdly, PS may improve the cognitive function through normalization the activity of NMDA receptor. NMDA receptor-mediated excitatory transmission is essential for the cognition function of the brain (Cohen and Muller, 1992). Compared to the young brains, the NMDA receptors in the aging brains alters, such as decreasing density of NMDA receptors and enhancing affinity to L-glutamine and glycine (Cohen and Muller, 1992). Chronic treatment with PS increased the density of NMDA receptors and normalized the affinity to L-glutamine and glycine in aging mice (Cohen and Muller, 1992). Fourthly, long-term potentiation requires a persistent increase in synaptic efficacy and is a critical synaptic mechanism of cognition (Thompson, 2000). Long-term potentiation needs the activation of NMDA receptor/channel complex. Treatment with exogenous PS also elicits synaptic efficacy (Borghese et al., 1993). Furthermore, exogenous PS stimulation also increased the metabolic levels of dopamine and serotonin which is lower in the cerebrospinal fluid in Alzheimer’s presenile dementia (Argentiero and Tavolato, 1980), and a recent study reported PS also increased the release of choline, which is an important neurotransmitter and decrease in AD brains (Suzuki et al., 2001), In summary, PS can improve the cognitive function of the brain through different pathways.

### Phosphatidylserine inhibits neuroinflammation in neurological diseases

Neuroinflammation is an immune response in the CNS and is mediated by microglia, astrocytes, or recruited macrophages (Woodburn et al., 2021). Neuroinflammation is involved in various conditions, including CNS injury, ischemia, infection, toxin, or autoimmunity (Woodburn et al., 2021). Neuroinflammation is a double-edged sword for the CNS:transient neuroinflammation may play a protective role during tissue repair after injury and remove the cellular debris; meanwhile, chronic neuroinflammation is related to the progression of CNS diseases such as AD, PD, stroke, and other brain injury (Catorce and Gevorkian, 2016; Leng and Edison, 2021).

Previous studies show that exogenous PS liposomes have an anti-inflammatory effect in the CNS (Bachiller et al., 2018). Treatment with PS liposomes or PS head group phospho-L-serine, the expression of pro-inflammation cytokines (such as TNFa and IL1β) and NO synthesis induced by LPS significantly decrease, and the expression of anti-inflammation cytokines (TGFβ and PGE2) significantly increase in microglia (De et al., 2002; Huynh et al., 2002; De Simone et al., 2003; De Simone et al., 2004; Zhang et al., 2006). Those studies suggested that PS liposomes may play a neuroprotective role through modulating microglia phenotype. PS and PC liposomes significantly increase the survival retinal neurons after I/R by reducing the expression of pro-inflammatory genes in microglia, such as IL1β, IL6, and C-C Motif Chemokine Ligand 2–5 (Dvoriantchikova et al., 2009). PS and PC liposomes also inhibit the microglial activation induced by Aβ and interferon-γ through reducing the production of TNFa, NO, and superoxide (Casamenti et al., 1991). Intranasal PS liposomes prior to surgical brain injury induction significantly increases TGFβ, and decreased IL1β and TNFa in brain tissue to attenuate inflammation (De Simone et al., 2004).

It is unknown how PS or its analogs mediates the anti-inflammation effects. Previous studies show that Mer, a PS receptor belonging to TAM receptor, triggered anti-inflammation of macrophages because Mer deficiency did not reduced LPS-induced inflammation in mice (Camenisch et al., 1999; Vago et al., 2021). CD36, another PS receptor, is also involved in anti-inflammation of PS liposomes (De Simone et al., 2004). A newly identified PS receptor-phosphatidylserine-specific receptor (PSR) may be involved in anti-inflammatory effect of PS (Fadok et al., 2001). Like other PS receptors, PSR is also involved in the phagocytosis but the more important function of PSR is to inhibit excess inflammation (Fadok et al., 2001). PSR in macrophages can inhibit the phagocytosis of apoptotic cells when PSR is bound to its ligands such as PS liposomes, phospho-L-serine, or PSR antibody; but promoted the anti-inflammation induced by LPS by increasing TGFβ and decreasing TNFa (Fadok et al., 2000). It remains unknown whether other PS receptors also are involved in this process. Mechanically, the interaction of the PS liposomes and PS receptor may inhibit several important inflammation regulators, such as p38 mitogen-activated protein kinase (p38MAPK), cyclic AMP responding element-binding protein (CREB), and NFκB. PS liposomes treatment significantly inhibits p38MAPK phosphorylation induced by LPS (Aramaki et al., 2001; Ajmone-Cat et al., 2003; Ma et al., 2011). Treating microglia with PS liposomes reduces phosphorylation of CREB induced by LPS (Ajmone-Cat et al., 2003). PS liposomes also inhibited the activation of NFκB induced by LPS (Aramaki et al., 2001; Ajmone-Cat et al., 2003). In addition, treatment with PS liposomes triggers the activation of ERK in microglia as early as 5 min (Ma et al., 2011). Therefore, the interaction between PS and PS receptors not only mediated the phagocytosis of apoptotic cells but also inhibit inflammation signaling and the release of anti-inflammation cytokines to have the anti-neuroinflammation effects.

## The roles of phosphatidylserine in different central nervous system diseases

As described above, a growing body of data implicate that both endogenous and exogenous PS plays critical roles in CNS diseases. Therefore, we summarized the current advances of PS in different CNS diseases, including AD, PD, MDD, ischemic stroke, ASD, and attention deficit hyperactivity disorder (ADHD).

### Alzheimer’s disease

Alzheimer’s disease AD is a progressive neurodegenerative disease; the pathological features of AD are characterized by the accumulation of amyloid-β (Aβ) plaques and phosphorylated tau neurofibrillary tangles (Paasila et al., 2021). There are many hypotheses about the mechanisms of AD, including synaptic dysfunctions hypothesis, cholinergic theory, amyloid cascade hypothesis, tau cascade hypothesis, neuroinflammation, and gut-brain axis hypotheses (Craig et al., 2011; Calsolaro and Edison, 2016; Kowalski and Mulak, 2019; Ju and Tam, 2022). Among these hypotheses, abnormal lipid metabolism in the cell membrane is considered as one of the mechanisms of AD. The alteration of PS and other phospholipids changes the viscosity of cell membrane and hinders many biological processes, such as enzyme activity, signal transduction efficiency, and membrane carrier (Akyol et al., 2021). It is controversary about the alternation of PS in brain tissues of AD patients. Some studies have demonstrated PS is reduced in brains of AD patients (Corrigan et al., 1998; Pettegrew et al., 2001; Oma et al., 2012; Sabogal-Guaqueta et al., 2020; Akyol et al., 2021). Meanwhile, others studies also found PS is increased or unchanged in brains of AD patients (Wells et al., 1995; Lampl et al., 2006; Martin et al., 2010; Kim W. S. et al., 2018). The details are shown in Table 2. Similar to AD patients, the changes of PS in brains are also not consistent in different animal models of AD as shown in Table 3 (Yao et al., 2009; Gonzalez-Dominguez et al., 2014; Martinez-Gardeazabal et al., 2017; Fitzner et al., 2020; Yi et al., 2020; Dejakaisaya et al., 2021). The reasons for this inconsistency may be explained by brain regions, Braak stages, age in the AD patients, and different experimental methods in studies. Therefore, PS is not considered as a robust diagnostic marker for AD (Tokuoka et al., 2019), but PS plays an important role in the mechanisms of AD. First, PS was reported to significantly increase the spine density of hippocampal pyramidal neurons (Nunzi et al., 1989; Spires-Jones et al., 2007); the reduction of dendritic spines is related to brain cognitive impairment in AD and the aged (Spires-Jones et al., 2007). Therefore, PS may ameliorate AD symptoms by restoring dendritic spines. Secondly, PS could negatively regulate the activity of PS synthase (PSS1 and PSS2) to prevent the depletion of PC and PE, resulting in high potassium-induced acetylcholine release (Casamenti et al., 1991; Suzuki et al., 2001; Bergo et al., 2002); the increase of acetylcholine release enhances the activity of cholinergic neurons and improve the cognitive function of AD patients. Thirdly, PS reduces the production of Aβ in CHO-APP/PS1 cells and Aβ-induced toxicity to primary hippocampal neurons (Xu et al., 2021). In addition, PS could combine with others drugs or function as a drug carrier to improve AD symptoms, for example, PS combines with ferulic acid and curcumin significantly to inhibit Aβ production, phosphorylated tau, and IL1β release, and increase brain-derived neurotrophic factor and acetylcholine (Okuda et al., 2019). PS also serves as drug delivery approach for metformin and nicotinamide to ameliorate the cognitive function and inflammation (Vakilinezhad et al., 2018; Saffari et al., 2020).

**TABLE 2:** The alteration of phosphatidylserine (PS) in Alzheimer’s disease (AD) patients.

Age (years)	Gender	Tissue	Method	PS content
81.33 ± 6.57	7M/8F	Neocortex	LC-MS/MS	Down
72.9 ± 0.8	–	Inferior parietal lobule Occipital cortex	^31^P NMR	Down
77.4 ± 7.2	3M/5F	Hippocampus	Gas chromatography	Down
76.5 ± 8.1	7M/9F	Erythrocyte Membrane	HPLC	Down
72.3 ± 10.6	4M/1F	Cortex	Annexin V SPECT imaging	Up
–	–	Hippocampus Temporal cortex	HPLC	Up
70.9 ± 5.7	8M/6F	Blood	LC-MS/MS	NA
81.2 ± 2.48	5M/5F	Cortex	HPTLC	NA
70.1 ± 16.3	4M/6F	White matter	ESI-MS/MS	Up
		Gray matter		Down
		Cerebrospinal fluid		Down

2D-HPLC, two-dimensional liquid chromatography/mass spectrometry;

HPLC, high performance liquid chromatography;

HPTLC, high performance thin layer chromatography;

^31^PNMR, ^31^P nuclear magnetic resonance.

**TABLE 3:** The alteration of phosphatidylserine (PS) in Alzheimer’s disease (AD) models.

Species	Model type	Age (month)	Tissue	Method	PS content
Mouse	APP/PS1	6	Hippocampus cortex	GC-MS	Up
Mouse	ApoE KO	20	Corpus callosum	MS	Up
Mouse	Tg2576	6	Cortex	LC-MS	Up
Rat	192IgG-saporin induced AD	–	Whole brain	IMS	Up
Mouse	APP/PS1	9	Brain cortex	HPLC	Down

Although the function of PS in AD has not been well clarified, clinical studies have shown that PS benefits patients with AD (Crook et al., 1992; Heiss et al., 1994; More et al., 2014). Clinical studies have shown that PS supplementation significantly improves cognitive function and memory loss in patients with AD. In a clinical trial, treatment the AD patients with PS (100 mg, three times a day for 12 weeks) significantly improved cognitive impairment, especially in the early stages of AD (Crook et al., 1992). Co-administration of 300 mg PS and 240 mg PA in AD patients also had shown the effects on emotion and daily life quality (More et al., 2014). Combination of cognitive training twice a week and PS treatment (200 mg, twice a day) in AD patients benefited brain functions and neuropsychological symptoms at 8 and 16 weeks after the treatment (Heiss et al., 1994). These studies indicated that PS could be used as a daily brain health supplement for AD patients.

### Parkinson’s disease

Parkinson’s disease is a neurodegenerative disorder, which clinically appears mainly as bradykinesia, rest tremor, and muscular rigidity (Israel and Hassin-Baer, 2005). Two pathogenesis features of PD are the loss of dopaminergic neurons and the presence of Lewy bodies in the substantia nigra and striatum. The degeneration of dopaminergic neurons leads to the lack of dopamine in the substantia nigra and striatum, while Lewy bodies contains a high concentration of α-Syn that are toxic to neurons (Witt, 2014). Recent studies have shown that abnormal lipid metabolism is also involved in the pathogenesis of PD. Phospholipid levels in peripheral blood of PD patients are higher than control subjects (Lobasso et al., 2017). It is found to have higher PS in the frontal cortex of PD at early stages (Fabelo et al., 2011; Canerina-Amaro et al., 2019). PS is also found to increased significantly in skin fibroblasts from Parkin-mutated PD patients (Li et al., 2015). Similar to PD patients, PS also increased in the brains of PD animal models (Canerina-Amaro et al., 2019). Therefore, PS is increased in the brain of PD patients and PD animals. However, the specific mechanism by which PS is involved in PD is still largely unknown and needs to be further studied.

Increasing PS was proved to promote the aggregation of α-Syn on phospholipid bilayers, which impairs the membrane permeabilization and may contribute to neuronal death in the substantia nigra in the brain of PD patients (Perrin et al., 2000; Zhao et al., 2004; Stockl et al., 2008; Lv et al., 2019; Hannestad et al., 2020). Kanamycin, an aminoglycoside antibiotic with positively charged amino groups, was reported to interfere with H-bonding between PS and α-Syn and inhibit aggregation of α-Syn on membrane (Mahapatra et al., 2019); thus, kanamycin may benefit PD patients. In addition, PS-riched exosomes also accelerated the aggregation of α-Syn and the transmission of α-Syn fibril between brain regions (Xia et al., 2019; Guo et al., 2020). Therefore, upregulated PS is related to the development of PD, and is a potential biomarker for the diagnosis of PD.

Interestingly, PS supplementation also benefits PD patients. In a double-blind study, PS administration showed a significant amelioration on some symptoms, such as motivation, anxiety, and affectivity in PD patients (Funfgeld et al., 1989). PS reversed memory impairment in reserpine-induced PD rat model (Alves et al., 2000); however, PS did not improve cognitive impairment in the classical MPTP-induced PD model (Perry et al., 2004), suggesting different mechanisms in reserpine/MPTP-induce memory impairment. Sleep disorders is a prodromal marker of PD (Tekriwal et al., 2017). In addition, PS can also serve as a drug delivery tool to elevate the bioavailability of drug, such as epigallocatechin-3-gallate and GDF5. Epigallocatechin-3-gallate, an antioxidant isolated from green tea with low bioavailability and high instability, is a potentially therapeutic for PD. Epigallocatechin-3-gallate–loaded PS liposomes reduced the production of nitric oxide, IL-1β, TNF-α, and COX induced by LPS *in vivo* and *in vitro* (Cheng et al., 2021). Furthermore, simultaneous intra-nigral injection of PS liposomes loaded with epigallocatechin-3-Gallate restored motor impairment in the rotation behavior test (Cheng et al., 2021). Similarly, PS liposomes loaded with growth differentiation factor GDF5 (a drug that can protect dopaminergic neurons from degeneration) with intranasal administration increased GDF5 concentration in the midbrain by 8 fold (Hanson et al., 2012). PS liposomes loaded with astragaloside IV and nestifin-1 facilitates the penetrating of the blood-brain barrier and reduced the expression of α-Syn (Kuo et al., 2021). Therefore, the PS liposome is a delivery tool for PD drugs.

### Major depressive disorder

Major depressive disorder is a very heterogeneous mental disorder. Genetic, psychological, and environmental factors are the main causes of MDD disease (Gu et al., 2020; Nemeroff, 2020; Cao et al., 2021). Previous studies showed that the concentration of PS in peripheral blood of MDD patients increased significantly compared with healthy controls (Kim E. Y. et al., 2018; Homorogan et al., 2021). The content of PS was found to increase in the rat brains of post-traumatic stress (Chaichi et al., 2021). In addition, escitalopram, an antidepressant drug, significantly reduces the concentration of PS in the peripheral blood of patients with MDD, and also improved depressive behaviors (Pastoor and Gobburu, 2014; Homorogan et al., 2021). Interestingly, supplementation with PS significantly improves depressive symptoms in both depressive animals and MDD patients. Clinical studies have reported that treatment of elderly MDD women with PS (200–600 mg/day) for 30 days significantly improved the depressive symptoms (Maggioni et al., 1990; Brambilla et al., 1996). Chronic PS administration (300 mg/day for 1–6 months) for MDD patients also reduced apathy and sleep disturbances, and increased motivation, and interest (Palmieri et al., 1987). Combined supplementation with 100 mg PS, 119 mg docosahexaenoic acid, and 70 mg eicosapentaenoic (three times a day) for 12 weeks significantly improved depressive behaviors in MDD patients, accompanying with the correction of the base level and circadian rhythm of salivary cortisol (Komori, 2015). In animal models, PS administration reduced the immobility time in the forced swimming test in mice, implicating the significant antidepressant effect of PS (Castilho et al., 2004). Treatment post-stroke depressive mice with PS liposomes significantly reduced the immobility time in the forced swimming test and tail suspension test (Partoazar et al., 2021). In addition, intracerebroventricular injection of PS also attenuated stress-induced behaviors in chick isolation-induced stress model (Koutoku et al., 2005).

The specific mechanism of antidepressant effects of PS is still largely unknown; A previous study demonstrated that co-injected PS with scopolamine, an antagonist of acetylcholine receptors abolished antidepressant effects of PS, indicating that muscarinic acetylcholine receptors are required for antidepressant effects of PS (Koutoku et al., 2005). Other studies have reported that PS treatment inhibits the production of ACTH, reduces the production of plasma cortisol, and then slows down the activation of the hypothalamus-pituitary-adrenal (HPA) axis in the process of MDD (Monteleone et al., 1992; Hellhammer et al., 2004; More et al., 2014). Although PS alleviated depressive behaviors in post-stroke depression mice through the reduction of pro-inflammatory cytokines such as TNF-α(Partoazar et al., 2021); the blood contents of IL1β, TNF-α, and IL6 did not alter in elderly patients with MDD after PS supplementation (Brambilla and Maggioni, 1998). Therefore, whether PS functions as an antidepressant through anti-inflammatory needs further studies or not. How PS improves depressive behaviors in MDD patients should be studied further.

### Ischemic stroke

Stroke is a very common and serious central nervous system disease that remains the second-leading cause of death and the third-leading cause of death and disability (Collaborators, 2021). Stroke leads to acute brain damage and cell death, which is accompanied by a series of physiological and biochemical changes, such as the increase of reactive oxygen species, calcium-dependent excitotoxicity, the alteration of electrolyte composition, the increase of cytochrome c released by mitochondria phospholipase mediated membrane damage (Fisher and Saver, 2015; Collaborators, 2021; Feske, 2021). PS has been demonstrated to be involved in the biological process of stroke. The content of PS was found to decrease significantly in the brain after ischemic injury; however, PS still decreased and remained below to control even after the long time reperfusion, although the contents of other lipids were restored quickly (Enseleit et al., 1984; Wieloch et al., 1991; Rao et al., 2000). The main reasons for the decrease of PS are unknown and may be related to a large number of cell deaths and the degradation of membrane structure after ischemic injury. The reduction of PS influences the activity of intercellular enzymes such as PKC (Huang et al., 2011). PKC is inactive in the cytosol and active once binds to PS in the presence of Ca^2+^ in the cell membranes; during ischemia and reperfusion, the total activity of PKC is reduced (Huang et al., 2011). The activity decreased of PKC was partly because PKC-α was dephosphorylated, transited from dimer to trimer, and lost the activity, while PKC-β is degraded by calpain (Louis et al., 1988; Wieloch et al., 1991; Harada et al., 1999). Those studies suggest that ischemia/reperfusion destroys the membrane system and changes the lipids in the membrane, so, the activity of the membrane-bound enzyme is decreased and normal function in the brain is impaired.

Similar to other diseases, PS also has a therapeutic effect for stroke. Ischemia/reperfusion injury has been demonstrated to elicit strong inflammatory responses mediated by activated microglia/macrophages. Microglia/macrophages can be activated by exposed PS on apoptotic cells (Zhao et al., 2017); however, PS liposomes can mimic apoptotic cells to target microglia/macrophages (Hosseini et al., 2015). PS modified microbubbles could cross the blood–brain barrier and target the activated microglia/macrophages in an ischemic stroke mouse model (Zhao et al., 2018). As described above, PS liposomes treatment promoted the production of anti-inflammatory factors, and inhibited the production of pro-inflammatory in phagocytes (Zhang et al., 2006). Therefore, PS liposomes may have a neuroprotective effect through enhancing the anti-inflammatory response of microglia/macrophages in stroke.

### Autism spectrum diseases

Autism spectrum disease is a neurodevelopmental disorder and is defined by communication and social deficit, coupled with repetitive and unusual sensory-motor behaviors (Mostafa et al., 2010). Genetic and environmental risk factors may contribute to ASD (Kim et al., 2019). The serum levels of PS were much lower in autistic patients than healthy subjects (El-Ansary et al., 2011). Another study found that serum PS levels decreased in autistic children with impaired sensory compared with control subjects (El-Ansary et al., 2016).

Autism spectrum disease is a heterogeneous neurodevelopment disease. Recently, the largest whole-exome sequencing study of ASD has identified 102 risk genes (Satterstrom et al., 2020). Although numerous biomarkers and risk genes have been reported, there is no robust biomarker to diagnose, prognosis, and predicted ASD. The reduction of PS in the blood of ASD patients could be a potential marker, but still need further studies to evaluate the effectivity in different subgroups.

### Attention deficit hyperactivity disorder

Attention deficit hyperactivity disorder is a neurodevelopmental disorder that is characterized by impairing inattention, impulsivity, and motor hyperactivity (Polanczyk et al., 2015). ADHD is a familial disorder and its genetic factors contribute to 76% (Thapar and Cooper, 2016). Several environmental factors are also linked to ADHD, ranging from prenatal and perinatal factors, dietary factors, environmental toxins, and psychiatric social factors (Mayer et al., 2021). First-line pharmacological treatments for ADHD are CNS stimulants, for example, methylphenidate and dexamfetamine. The second-line treatment is the noradrenaline reuptake inhibitor atomoxetine (Thapar and Cooper, 2016). However, 20–30% of children has failed to respond to those drugs or cannot tolerate (Mayer et al., 2021). A clinical study reported that treatment ADHD children with PS-omega3 (250mg/day) for 30 weeks significantly improved ADHD symptoms including hyperactive-impulsive, emotionally, and behavioraly-dysregulated symptoms (Manor et al., 2012). Another clinical observation also showed that supplementation with 200 mg/day PS for 2 months resulted in significant improvements in overall symptoms and short-term auditory memory in ADHD children (Hirayama et al., 2014). However, it is also showed that treatment ADHD patients with 200–300?mg/day of PS significantly reduced inattention, while no effects on overall symptoms of ADHD and hyperactivity-impulsivity (Bruton et al., 2021). In conclusion, PS supplement serves as a no adverse effect and natural nutritional strategy for improving symptoms and ADHD patient’s quality of life (Manor et al., 2013). ADHD is a high prevalence mental disorder and affects a whole lifetime (Polanczyk et al., 2015), whether PS also works in adult ADHD patients need further studies.

### Schizophrenia

Schizophrenia is a mental disorder described by impairment of cognitive, behavior, and motion. Symptoms of schizophrenia include delusions, disorganized speech, and hallucinations (Tandon et al., 2013). PS was increased in the thalamic (Schmitt et al., 2004), and not changed in hippocampus of patients with schizophrenia (Hamazaki et al., 2010), although thalamic and hippocampus both are critical brain regions for cognition, perhaps they are contributed to schizophrenia through different mechanisms. On the contrary, the level of PS is lower in fibroblasts and red blood cell from schizophrenia patients (Mahadik et al., 1994; Ozcan et al., 2008), however, alteration of PS in red blood cell membrane of schizophrenia patients is inconsistent in different studies, several studies also supports that PS is not change (Lautin et al., 1982). Abnormal membrane phosphatidylserine and other lipids of fibroblast and red blood cell may predate the onset of schizophrenia (Lautin et al., 1982), could sever as predicted biomarker. PS is also related to poor response of antipsychotics treatments in schizophrenia patients, lower level of PS is found in poor responses to risperidone, olanzapine, and quetiapine (de Almeida et al., 2020), due to the important role of PS in brain function, lower PS may be involved in worse outcomes of treatment.

### Spinal cord injury

Spinal cord injury (SCI) is serious CNS disease which caused by traumatic and non-traumatic reasons, and often leads to impairments of sensory and motor function (Anjum et al., 2020). Reduction of PS in spinal cord is reported in several SCI animal models, including experimental autoimmune encephalomyelitis (Chevalier and Rosenberger, 2017), ischemia/reperfusion, and experimental traumatic injury (Lukacova et al., 1996). These studies support that SCI accompanied with the rapid degradation of PS and difficult to restore. In ischemia/reperfusion injury, PS is decreased during ischemia, and only partly region of spinal cord restore to control even after 3 hours long term reperfusion (Lukacova et al., 1996). PS is the main component of myelin sheath, and loss of myelin sheath is common accompaniment of SCI, so to increase PS may benefit SCI patients (Horrocks, 1973; Waxman, 1992; Anjum et al., 2020). Glyceryl triacetate treatment significantly increased PS in experimental autoimmune encephalomyelitis mice model, and improved the loss of myelin (Chevalier and Rosenberger, 2017).

## Conclusion

Phosphatidylserine is important nutritional component in the cell membrane, especially with high proportion in the brain and has critical multiple functions involving in cellular signal transduction, cell death and survival, and inflammation. As we summarize in Table 4, alterations of PS are observed in the serum and the brains in different CNS diseases, but the specific biological effects of altered PS in different CNS diseases remain largely unknown and warrant further investigations. However, a body of evidence showed that oral PS benefits patients with different CNS diseases including AD, PD, MDD, and ADHD. In addition, clinical studies also showed that PS had no side effects and was well tolerated (Heiss et al., 1994). Therefore, PS and PS liposome could be a promising supplementation for these neurodegenerative and neurodevelopmental diseases.

**TABLE 4:** The trend and function of phosphatidylserine (PS) in central nervous system (CNS) disease.

Disease	Trend of PS		Function of PS	PS improves disease
	
	Patient	Animal model		
AD	Inconsistent	Inconsistent	Increase dendritic spine, increase acetylcholine, inhibit the microglial activation and tau hyperphosphorylation	Yes
PD	Up	Up	Initiate and enhance aggregation of α-Syn, function as carrier to deliver other drugs	Yes
MDD	Up	Up	Blunt the activation of hypothalamus pituitary adrenal axis, attenuate the cytotoxicity of corticosterone	Yes
Stroke	No data	Down	Reduced PS decrease the activity of PKC, PS liposomes inhibit inflammation	No data
ASD	down	No data	No data	No data
ADHD	No data	No data	No data	Yes
Schizophrenia	inconsistent	No data	No data	No data
SCI	No data	Down	No data	No data

## Author contributions

XM and XL were responsible for writing and literature review. MZ and WW were responsible for drawing the picture. BY and ZM were responsible for the conception and editing of the article. All authors contributed to the article and approved the submitted version.
